# Examining differences in phylogenetic composition enhances understanding of the phylogenetic structure of the shrub community in the northeastern Qinghai‐Tibetan Plateau

**DOI:** 10.1002/ece3.6402

**Published:** 2020-06-08

**Authors:** Yuanming Xiao, Lucun Yang, Xiuqing Nie, Changbin Li, Feng Xiong, Lingling Wang, Guoying Zhou

**Affiliations:** ^1^ Northwest Institute of Plateau Biology Chinese Academy of Sciences Xining China; ^2^ University of Chinese Academy of Sciences Beijing China; ^3^ Key Laboratory of Tibetan Medicine Research Chinese Academy of Sciences Xining China; ^4^ Qinghai Key Laboratory of Qinghai‐Tibet Plateau Biological Resources Xining China

**Keywords:** climatic oscillations, phylogenetic community composition, phylogenetic community structure, Qinghai‐Tibetan Plateau, shrub, species dispersal

## Abstract

Periodic climatic oscillations and species dispersal during the postglacial period are two important causes of plant assemblage and distribution on the Qinghai‐Tibet Plateau (QTP). To improve our understanding of the bio‐geological histories of shrub communities on the QTP, we tested two hypotheses. First, the intensity of climatic oscillations played a filtering role during community structuring. Second, species dispersal during the postglacial period contributed to the recovery of species and phylogenetic diversity and the emergence of phylogenetic overdispersion. To test these hypotheses, we investigated and compared the shrub communities in the alpine and desert habitats of the northeastern QTP. Notably, we observed higher levels of species and phylogenetic diversity in the alpine habitat than in the desert habitat, leading to phylogenetic overdispersion in the alpine shrub communities versus phylogenetic clustering in the desert shrub communities. This phylogenetic overdispersion increased with greater climate anomalies. These results suggest that (a) although climate anomalies strongly affect shrub communities, these phenomena do not act as a filter for shrub community structuring, and (b) species dispersal increases phylogenetic diversity and overdispersion in a community. Moreover, our investigation of the phylogenetic community composition revealed a larger number of plant clades in the alpine shrub communities than in the desert shrub communities, which provided insights into plant clade‐level differences in the phylogenetic structures of alpine and desert shrub communities in the northeastern QTP.

## Graphical abstract

In this manuscript, we studied the phylogenetic community composition of shrub in the northeastern Qinghai‐Tibetan Plateau and found that existing significant difference in phylogenetic community composition of shrub communities from different habitats (desert and alpine), which interpreted phylogenetic community structure from perspective of phylogenetic community composition, enhanced our knowledge of shrub, and helped us to obtain further interpretation for present‐day community structure from historical processes.

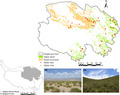

## 1 INTRODUCTION

Phylogenetic‐based community ecology, which incorporates the evolutionary relationships between species, has facilitated our understanding of the mechanisms underlying the structures of plant assemblages (Aldana, Carlucci, Fine, & Stevenson, 2017; Liu, Che, Jiao, Li, & Jiang, 2019; Swenson et al., 2012; Webb, 2000; Webb, Ackerly, McPeek, & Donoghue, 2002; Zhou et al., 2018; Zu et al., 2019). Approaches in this field enable community ecologists to identify patterns of species clustering or overdispersion in a local community and associate these with short‐term local ecological processes (Webb, 2000; Webb et al., 2002). Recent studies have applied community phylogenetic approaches to paleo‐geologic historical conditions (e.g., climatic oscillations and species dispersal) to investigate existing plant communities (Aldana et al., 2017; Feng et al., 2014). For example, Feng et al. (2014) observed that climatic oscillations strongly affected the phylogenetic community structures of Chinese forests.

The Qinghai‐Tibet Plateau (QTP) is the highest and largest plateau in the world (average elevation, >4,000 m; area, 2.5 × 10^6^ km^2^) and features a typical high‐altitude ecosystem (Zhang, Li, & Zheng, 2002). To date, however, only a few studies have examined the distribution patterns of phylogenetic diversity and the phylogenetic community structures of plants, and particularly shrub vegetation, in this area (Li & Sun, 2017; Xiao et al., 2018; Yan, Yang, & Tang, 2013). Shrubs are among the main vegetation types on the QTP (accounting for an approximate area of 10.98 × 10^4^ km^2^ on the plateau) and play vital ecological roles (Nie, Xiong, Yang, Li, & Zhou, 2017; Noumi, Chaieb, Le Bagousse‐Pinguet, & Michalet, 2016).

The uplift of the QTP induced significant changes in the climatic oscillations of the region (Wen, Zhang, Nie, Zhong, & Sun, 2014) and thus had important and direct effects on plant community structuring and distribution (Mosbrugger, Favre, Muellner‐Riehl, Päckert, & Mulch, 2018). The possibility that species that were unable to adapt to these changes may have been eliminated from the community suggests that climatic oscillations play an important filtering role. Feng et al. (2014) showed that a plant community tends to exhibit phylogenetic clustering in response to strong climatic oscillations, whereas milder climatic changes induce higher phylogenetic diversity and phylogenetic overdispersion. Therefore, we were interested to determine whether paleoclimatic oscillations played a filtering role in the assemblage of the shrub community on the QTP, which has exhibited increased phylogenetic clustering since the intensification of paleoclimatic oscillations.

Species dispersal during the postglacial period is another vital aspect of plant community structuring and distribution on the QTP (Wen et al., 2014; Yu et al., 2017). Yu et al. (2017) reported that mountains and valleys (e.g., the Himalayas and Yarlung Zangbo Valley) served as corridors for species migration and dispersal and formed three major species dispersal routes. We expected that the phylogenetic diversity of a community could recover rapidly from the possible filtering effect of a cold snap in a species dispersal area. In particular, the dispersal of distantly related species to these regions may have greatly increased the regional phylogenetic diversity, leading to phylogenetic overdispersion (Carlucci et al., 2017). Indeed, lower levels of species dispersal are observed in desert regions than in alpine/mountain regions (Yu et al., 2017). Therefore, we hypothesized that species dispersal during the postglacial period contributed to the recovery of species and phylogenetic diversity, leading to phylogenetic overdispersion in alpine/mountain regions. These changes may have more effectively increased the phylogenetic diversity of shrub communities in alpine regions than in desert regions, which would be accompanied by stronger overdispersion of the phylogenetic community structure in the former regions.

In this study, we investigated 61 shrub sites mainly distributed in the desert and alpine/mountain regions of the northeastern QTP (Qinghai Province), which provided an ideal opportunity to test our above hypotheses. Additionally, we generated principal coordinates of the phylogenetic structure (PCPS) (Debastiani & Duarte, 2014; Duarte, 2011) to provide straightforward evidence of the phylogenetic community composition underlying this structure in the shrub communities on the northeastern QTP.

## 2 MATERIALS AND METHODS

### 2.1 Study area

**FIGURE 1:**
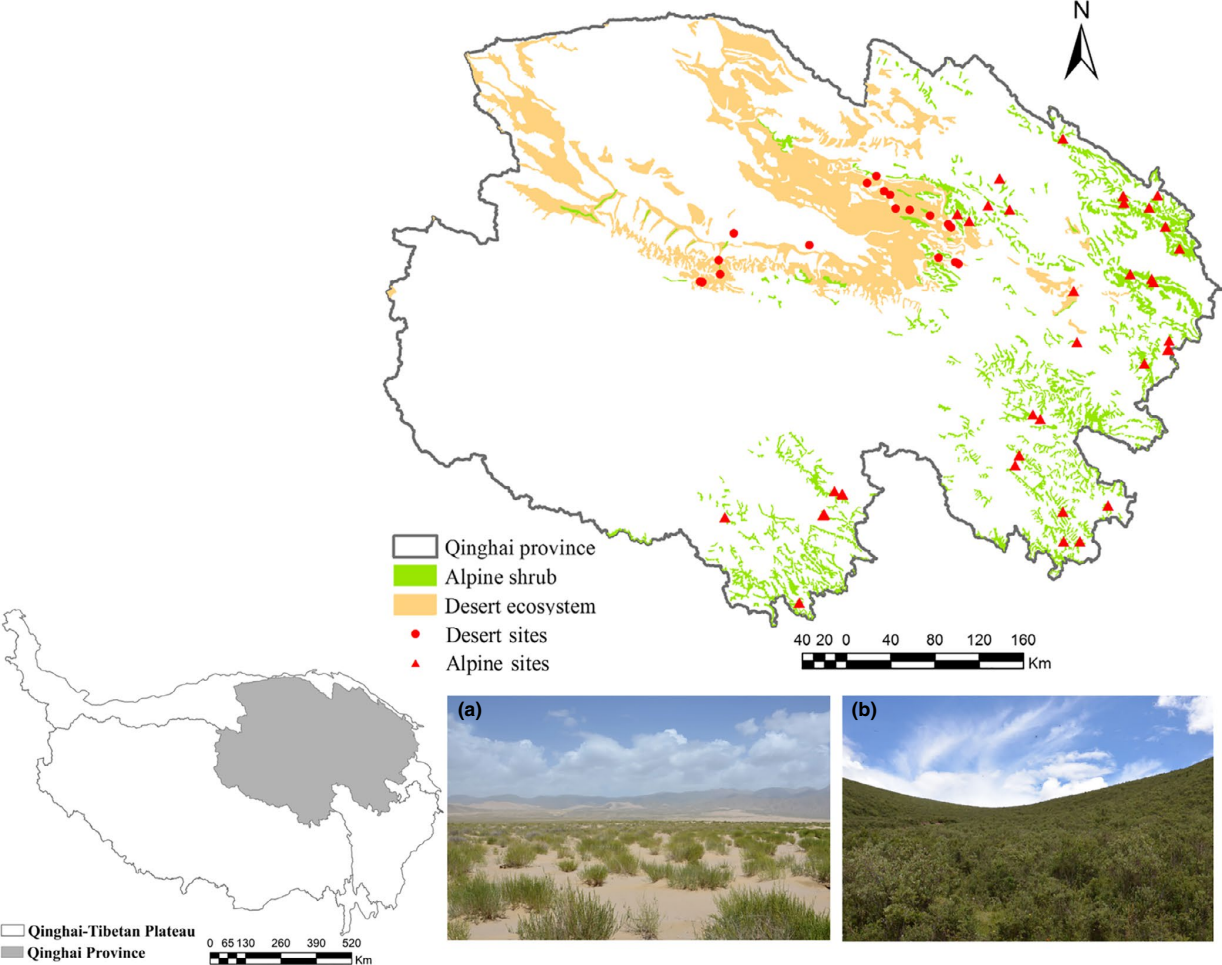
Locations of the 61 sampling sites on the northeastern Qinghai‐Tibetan Plateau (Qinghai Province, China). The circles and triangles in red represent sampling sites in the desert and alpine habitats, respectively, and comparisons were made between these habitats. Representative images of the desert habitat (a) and alpine habitat (b). All photographs were taken by GuoYing Zhou

The sampling sites were located on the northeastern QTP (Qinghai Province, 89°35′–103°04′E, 31°9′–39°19′N), which has an average elevation, mean annual temperature range, and mean annual precipitation range of > 3,000 m above sea level, −3.7 to 6.0°C, and 17–776 mm, respectively (Zheng & Zhao, 2017). Our shrub sampling sites were distributed across alpine and desert shrub habitats (Figure 1). The desert shrub sites were distributed throughout the Qaidam Basin, an area in which the community is characterized by a relatively simple species composition and a low species diversity. The main species are *Tamarix hohenackeri*, *Haloxylon ammodendron*, *Ceratoides latens,* and *Nitraria tangutorum*. In contrast, the alpine shrub sites were distributed across areas other than the Qaidam Basin and were characterized by a relatively high species diversity. The main alpine species included *Sibiraea laevigata*, *Salix oritrepha*, *Rhododendron capitatum,* and *Caragana jubata*. Although the desert shrub data were published previously by Xiao et al. (2018), a comparison between the desert and alpine shrub communities remains a very meaningful strategy for elucidating the processes and mechanisms underlying shrub differences between the two habitats.

We surveyed 20 sampling sites from the desert habitat and 41 from the alpine habitat with little or no disturbance (total = 61; Figure 1). Three 10‐m × 10‐m subplots were established at each sampling site to investigate the species composition of the community, identify species, and collect plant specimens. Eventually, a combination of the species found in the three subplots was used to represent the species composition of the community at each site.

### 2.2 Phylogenetic tree

We constructed a phylogenetic tree to present the evolutionary relationships between the 285 species found at our sampling sites. As in many previous studies (Barak et al., 2017; Zhou et al., 2018; Zu et al., 2019), we used the megatree containing 32,223 plant species published by Zanne et al. (2014) as a backbone and used the Phylomatic platform (Webb & Donoghue, 2005) to prune this megatree to include only the species found at our sampling sites. In this study, we only focused on angiosperms.

### 2.3 Paleoclimatic factors and phylogenetic community structure and composition

To measure the effects of climatic oscillations on the shrub communities, we used the mean annual temperature (MAT) anomaly (current MAT to paleoclimate MAT) to represent the climatic oscillations (Feng et al., 2014). We established three MAT anomalies ranging from the Last Glacial Maximum (LGM)‐MAT to the current MAT, from the mid‐Holocene (MH)‐MAT to the current MAT and from the LGM‐MAT to the MH‐MAT.

To identify differences in phylogenetic diversity, we used the PD index, which quantifies the phylogenetic diversity in a community and represents the total phylogenetic branch length spanned by all species in a community (Faith, 1992). We assessed differences in the phylogenetic community structures of shrub communities between the two habitats using the net relatedness index (NRI) and nearest taxon index (NTI) (Webb et al., 2002), which indicate the mean phylogenetic distance (MPD) between any possible pairwise species and the mean nearest taxon distance (MNTD) between any species and its nearest relative species within a community, respectively (Webb et al., 2002). Before calculating these indexes, we defined our species pool using the species found at all sampling sites. The NRI and NTI were determined using the following formulas:

$$\text{NRI}=-1\frac{\left[{\text{MPD}}_{\text{obs}}-{\;}_{\text{mean}}\left({\text{MPD}}_{\text{null}}\right)\right]}{{\;}_{\text{SD}}\left({\text{MPD}}_{\text{null}}\right)}$$

$$\text{NTI}=-1\frac{\left[{\text{MNTD}}_{\text{obs}}-{\;}_{\text{mean}}\left({\text{MNTD}}_{\text{null}}\right)\right]}{{\;}_{\text{SD}}\left({\text{MNTD}}_{\text{null}}\right)}$$

where *MPD*_obs_ and *MNTD*_obs_ represent the observed MPD and MNTD of the community; _mean_(*MPD*_null_) and _mean_(*MNTD*_null_) represent the mean MPD and MNTD expected by chance, for which species were drawn 999 times from the species pool while using the same probability and maintaining the same species richness; and *_SD_*(*MPD*_null_) and *_SD_*(*MNTD*_null_) are the standard deviations of the MPD and MNTD expected by chance. A positive NRI or NTI indicates that a community comprises closely related species and has a clustered phylogenetic structure. Conversely, a negative NRI or NTI indicates that the species in a community are more distantly related than would be expected by chance and that the phylogenetic community structure is overdispersed. An NRI or NTI close to 0 or that differs nonsignificantly from 0 indicates no significant phylogenetic community structure (e.g., phylogenetic randomness) (Webb, 2000; Webb et al., 2002).

Next, we determined the presence of differences in the phylogenetic community compositions of the shrub communities between the two habitats. To this end, we used PCPS (Debastiani & Duarte, 2014; Duarte, 2011) to identify major changes in the phylogenetic community composition and demonstrate the associations between the clade and site distributions across a set of communities. PCPS are ordination vectors obtained through a principal coordinates analysis of a matrix P of the phylogeny‐weighted species composition of all communities (Duarte, 2011; Duarte, Debastiani, Freitas, & Pillar, 2016; Seger et al., 2017). The PCPS with the highest eigenvalue indicates a major difference in the phylogenetic community composition (Carlucci et al., 2017). According to Maestri et al. (2016), the PCPS method is not strongly affected by the resolution at the tip nodes of the phylogenetic tree. In our study, we calculated the PD, NRI, and NTI using the *Picante* (Kembel et al., 2010) package and the PCPS using the *PCPS* (Debastiani & Duarte, 2014) package in the R environment (R Development Core Team, 2015).

### 2.4 Statistical analysis

First, we used variation partitioning to determine the effects of climatic oscillations on the shrub communities. Next, we used the Wilcoxon test to determine significant differences in phylogenetic diversity, phylogenetic community structure, and the values of the first PCPS axes (PCPS I scores) between the two types of shrub communities (Chai et al., 2018). We used Pearson's correlation analysis to compute the correlation coefficients of the phylogenetic community structure index with the first and second PCPS axes (PCPS I and II scores, respectively, as shown in the Appendix S1) and with environmental factors. All of the statistical analyses were performed in the R environment (R Development Core Team, 2015).

## 3 RESULTS

### 3.1 Differences in species diversity, phylogenetic diversity, and phylogenetic community structures between the two habitats

**FIGURE 2:**
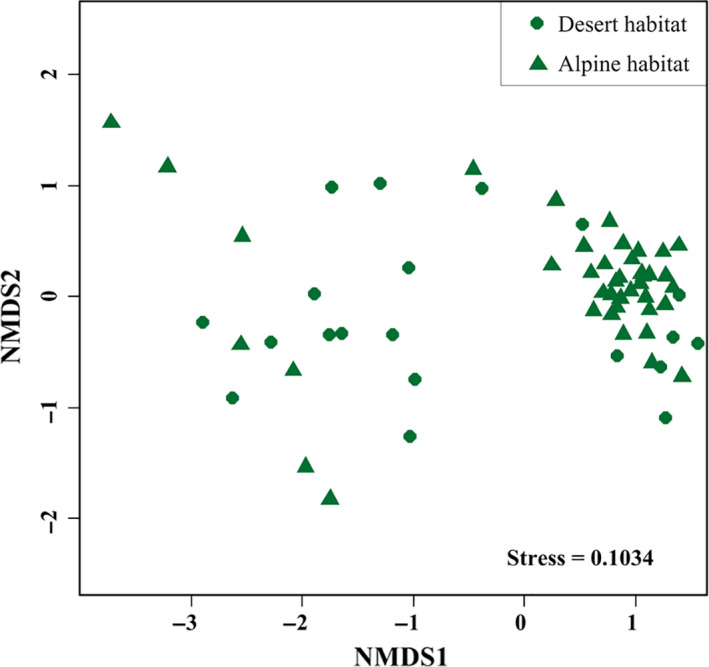
Nonmetric multidimensional scaling (NMDS) ordinations of the species compositions of the shrub communities in the desert (circle) and alpine (triangle) habitats on the northeastern Qinghai‐Tibet Plateau

**FIGURE 3:**
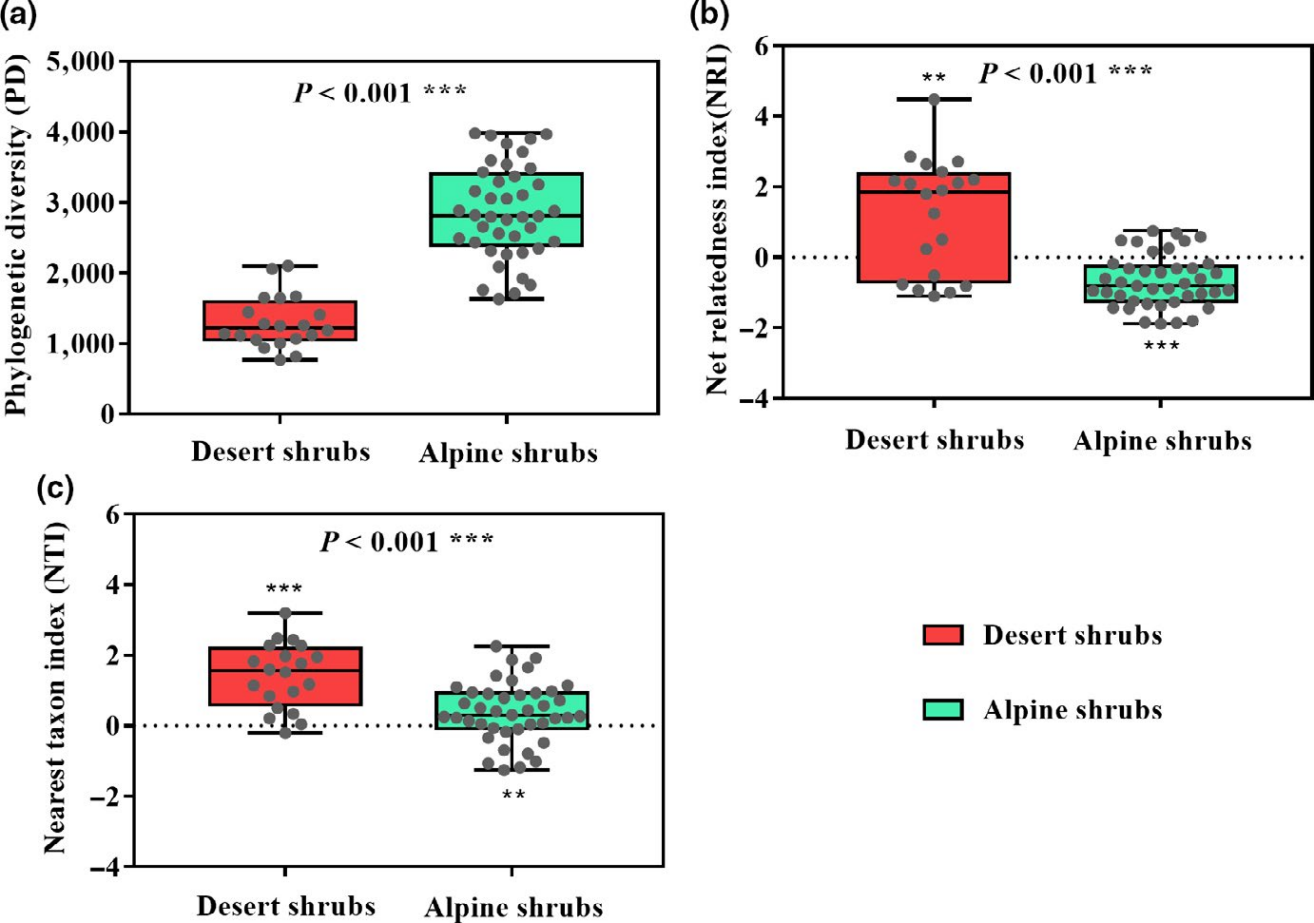
Distributions of the phylogenetic diversity (a), net relatedness index (NRI) (b), and nearest taxon index (NTI) (c) within the desert and alpine shrub communities on the northeastern Qinghai‐Tibet Plateau. The Wilcoxon test was used to determine the significant differences. NRI or NTI values of >0 and <0 indicated a clustered and overdispersed phylogenetic community structure, respectively. The asterisks in (b) and (c) indicate that the mean NRI or NTI value differed from 0: **p < .01 and ***p < .001. The significant differences between NRI, NTI, and 0 were examined using Student's *t* test. All data are indicated by gray dots in the boxplot

A total of 285 angiosperm species belonging to 45 families and 158 genera were identified at the 61 shrub sites in our study (Figure 1; Table S1). Differences were identified between the two habitats in terms of the species compositions of the shrub communities (Figure 2), especially species richness, which was significantly higher in the alpine habitat (33.39 species per plot) than in the desert habitat (12.35 species per plot) (Figure S2). The phylogenetic diversity was also significantly higher in the alpine habitat than in the desert habitat (Figure 3a).

In the desert habitat, most NRI and NTI values were >0, indicating that the phylogenetic community structure was clustered (Table 1, Figure 3b,c). In contrast, in the alpine habitat, most NRI values were <0, indicating that the phylogenetic community structure was overdispersed (Table 1, Figure 3b). However, most NTI values in the alpine habitat were >0 but only 9.76% were significantly >0, indicating that the phylogenetic structure was random (Table 1, Figure 3c). A higher degree of phylogenetic clustering was observed in the desert shrub community than in the alpine shrub community (Figure 3b,c).

**TABLE 1:** Numbers of sampling sites in the two shrub habitats and distribution ranges of the net relatedness index (NRI) and nearest taxon index (NTI) values

Shrub type	No. of sampling sites	NRI < 0	NRI < 0 (*p* < .05)	NRI > 0	NRI > 0 (*p* < .05)	NTI < 0	NTI < 0 (*p* < .05)	NTI > 0	NTI > 0 (*p* < .05)
Desert shrub	20	6 (30.00%)	0 (0.00%)	14 (70.00%)	11 (55.00%)	1 (5.00%)	0 (0.00%)	19 (95.00%)	10 (50.00%)
Alpine shrub	41	33 (80.49%)	10 (24.39%)	8 (19.51%)	0 (0.00%)	11 (26.83%)	0 (0.00%)	30 (73.17%)	4 (9.76%)
Overall	61	39 (63.93%)	10 (16.39%)	22 (36.07%)	11 (18.03%)	12 (19.67%)	0 (0.00%)	49 (80.33%)	14 (22.95%)

### 3.2 Determinants of the phylogenetic diversity and phylogenetic structure of a shrub community

**FIGURE 4:**
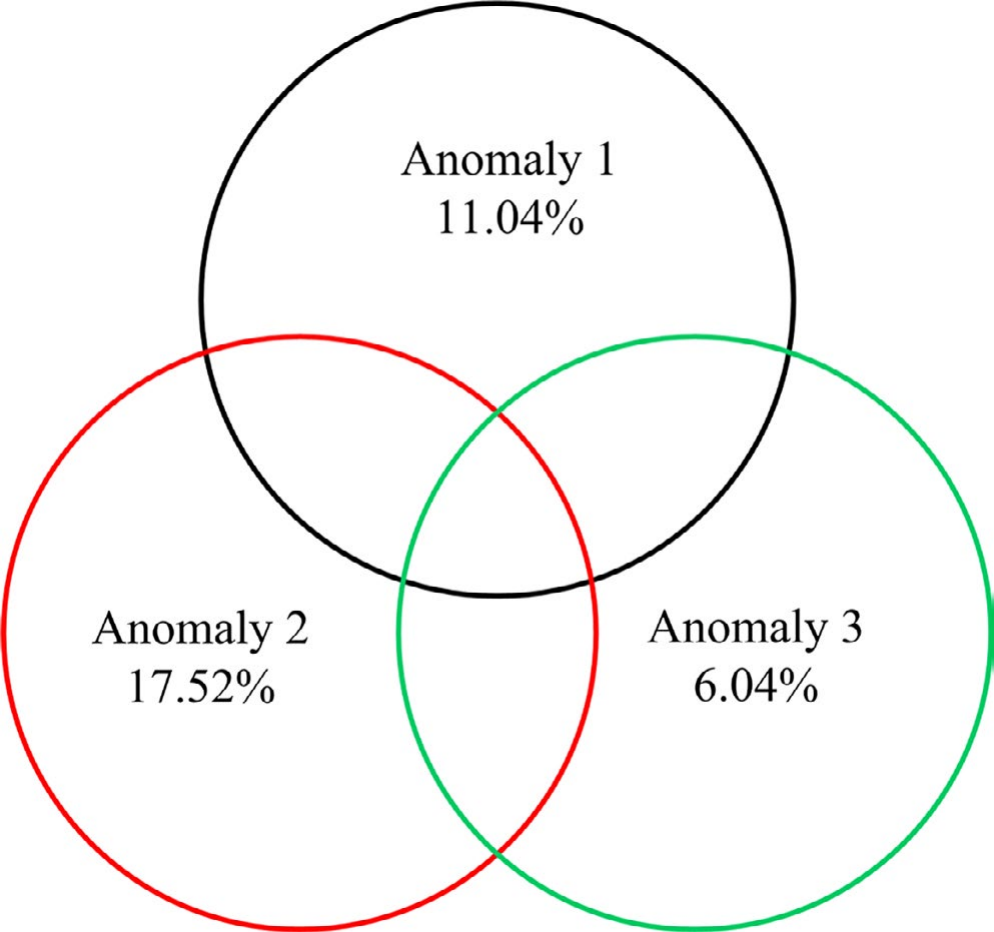
Partitioning of the variations in phylogenetic diversity attributed to Anomaly 1, Anomaly 2, and Anomaly 3. These anomalies represent three periods of climatic oscillations, namely from the current mean annual temperature (MAT) to the mid‐Holocene MAT, from the current MAT to the last glacial maximum MAT and from the mid‐Holocene MAT to the last glacial maximum MAT, respectively

**FIGURE 5:**
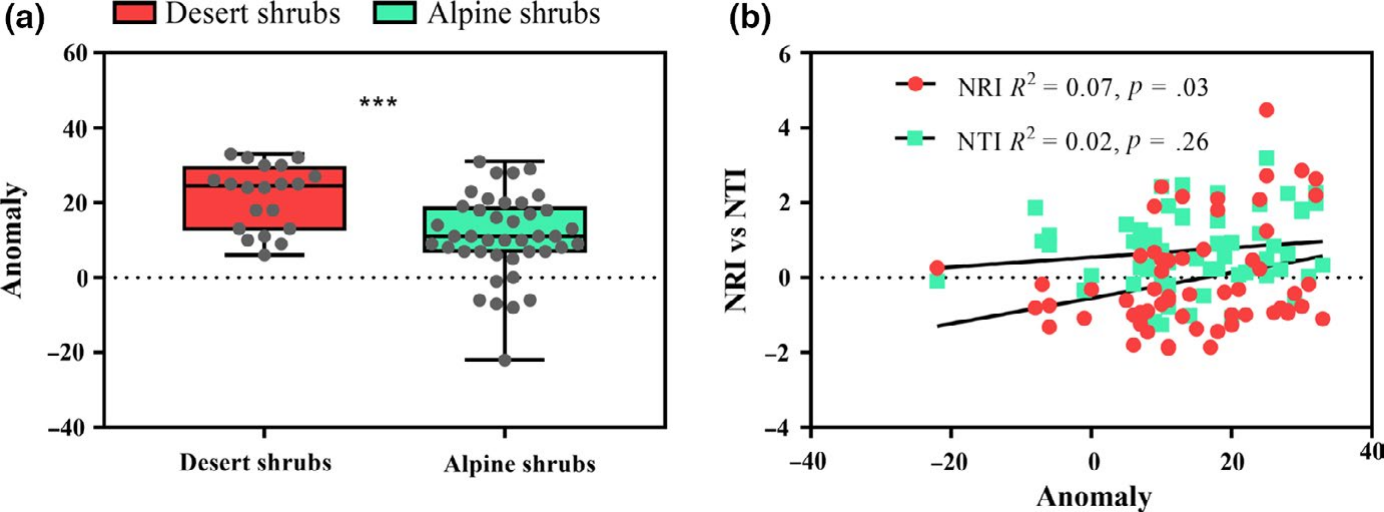
Anomaly 2 (from the current MAT to the last glacial maximum MAT) in the alpine and desert habitats (a). Significant differences were determined using the Wilcoxon test. All data are represented by gray dots. The relationships of the NRI and NTI with Anomaly 2 are shown in (b)

Our results revealed that the paleoclimatic oscillations and environmental factors affected the phylogenetic diversity and phylogenetic structures of the shrub communities. We determined that the MAT anomaly comprising the current MAT to LGM‐MAT had the highest explanatory power for the PD index of the shrub communities (Figure 4) and that this anomaly was stronger in the alpine habitat than in the desert habitat (Figure 5a). Meanwhile, the NRI decreased significantly as this anomaly increased (Figure 5b).

The PD index was correlated negatively with the MAT, but strongly positively with the other environmental factors. The NRI was correlated positively with the MAT, but negatively with the other environmental factors. Furthermore, the NTI exhibited negative correlations with only the mean annual precipitation (MAP), soil water content (SWC), and coverage and no correlations with the other environmental factors (Table 2).

**TABLE 2:** Pearson's correlation analysis of the associations of phylogenetic diversity (PD) and the phylogenetic community structure [net relatedness index (NRI) and nearest taxon index (NTI)] with climatic and environmental factors

	Anomaly 2	MAT	MAP	SWC	SR	Coverage
PD	0.35*	−0.52*	0.64*	0.46*	0.98*	0.31*
NRI	−0.30*	0.60***	−0.50*	−0.56*	−0.58*	−0.32*
NTI	−0.18 ns	0.23 ns	−0.53*	−0.50*	−0.21 ns	−0.48*

Abbreviations: coverage, canopy coverage of shrubs; MAP, mean annual precipitation; MAT, mean annual temperature; SR, species richness; SWC, soil water content.

* *p* < .05, **p < .01 and **p < .001; ns, nonsignificant.

### 3.3 Differences between the two habitats in terms of the phylogenetic compositions of the shrub communities

**FIGURE 6:**
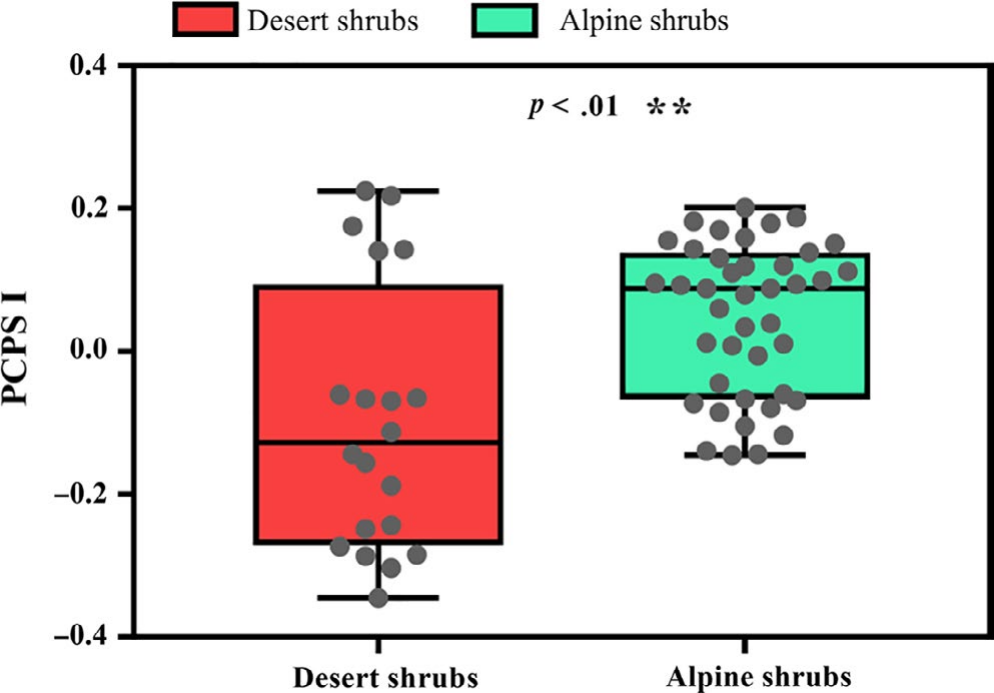
Distribution of the principal coordinates of the phylogenetic structure (PCPS) I scores between the desert and alpine shrub communities on the northeastern Qinghai‐Tibetan Plateau. The significant difference was determined using the Wilcoxon test. All data are represented by gray dots

**FIGURE 7:**
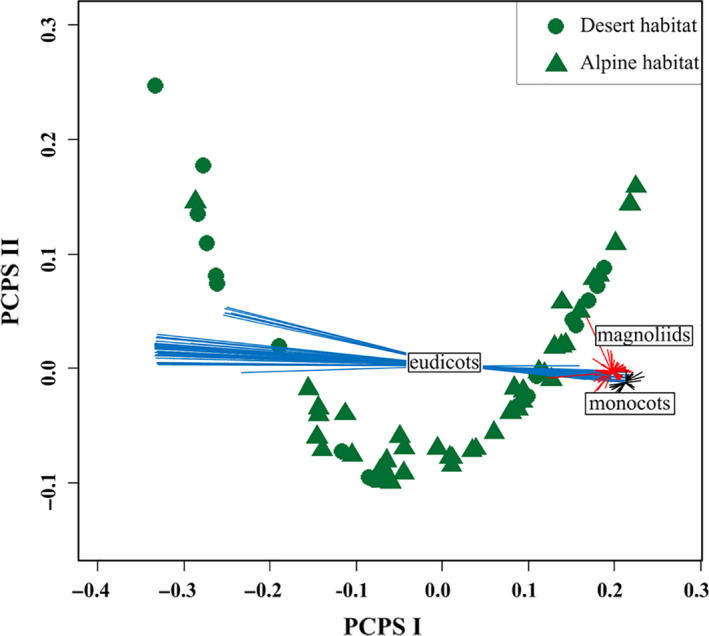
Scatter plot of variations in the phylogenetic community compositions across the desert shrub communities (circles) and alpine shrub communities (triangles). The spider‐like diagrams indicate the positions of species relative to the centroids of their clades in the multivariate space

Pearson's correlation analysis revealed a stronger correlation between the NRI and PCPS I than between the NRI and PCPS II (Table S2). Significant differences in the PCPS I scores were observed between the desert and alpine shrub communities (Figure 6), indicating a significant difference in the phylogenetic compositions of these communities. Further, the PCPS revealed that eudicots represented a larger percentage of the phylogenetic composition in the desert shrub community, whereas eudicots, monocots, and magnoliids were all well represented in the alpine habitat (Figure 7).

## 4 DISCUSSION

### 4.1 Phylogenetic diversity and phylogenetic structures of the shrub communities in the two habitats

We observed significant differences in the phylogenetic diversity and phylogenetic community structures when we compared shrubs between the alpine and desert habitats (Figures 2 and 3). Specifically, the degree of divergence in the phylogenetic community structure was stronger in the alpine habitat than in the desert habitat (Figure 3), suggesting that species in the alpine shrub community were more distantly related than were those in the desert shrub community. Although the uplift of the QTP and topography‐induced variations have important effects on the plant communities (Wen et al., 2014), climate change is thought to be the true driver of these effects (Mosbrugger et al., 2018). Therefore, periodic climatic oscillations may be a vital cause of the observed differences in the shrub communities between the two habitats on the QTP (Figures 4 and 5).

We determined that the paleoclimatic anomaly representing the current MAT to the LGM‐MAT had the strongest effect on the shrub communities (Figure 4). This observation suggests that the degree of divergence in the phylogenetic community structure increases as the paleoclimatic anomaly increases (Figure 5b). This result did not support our first hypothesis, namely that the intensity of climate anomaly would not play a strong filtering role in the shrub community assemblage. Feng et al. (2014) reported that in a forest community, a milder paleoclimatic anomaly helped to preserve greater phylogenetic diversity, leading to phylogenetic overdispersion. These contradictory results may be attributable to significant differences in the types of organisms between the forest and shrub communities (Yan et al., 2013). In a shrub community, the current climatic factors have a stronger effect than the paleoclimatic oscillations (Table 2). Thus, these significant differences in environmental factors between the alpine and desert habitats (Figure S3) largely explain the results of our study. Particularly, we can predict that the species composition and phylogenetic diversity in a desert shrub community would be more diverse and greater, respectively, based on the close negative correlations of these communities with water‐related factors, according to the situation involving a gradual increase in precipitation on the QTP (Yang, 2018).

Species dispersal in the postglacial period has had important effects on the plant community (Wen et al., 2014; Yu et al., 2017). As noted above, Yu et al. (2017) reported three major species dispersal routes through the mountains and valley, which would have been expected to enable the rapid recovery of species diversity and phylogenetic diversity in communities during the postglacial period (Wen et al., 2014). Our results are consistent with the hypothesis that species dispersal during the postglacial period contributed to the recovery of species and phylogenetic diversity, as well as phylogenetic overdispersion.

In addition to species dispersal, glacial refugia, which have a complex topography and geomorphology, significantly affected the recovery of local species diversity and phylogenetic diversity (Ye et al., 2018; Yu et al., 2015, 2019). For example, some studies reported higher levels of phylogenetic diversity in the Hengduan Mountain region than in the surrounding areas (Li & Sun., 2017; Yan et al., 2013). Accordingly, the refugia in the regions of three river sources (Yang, Li, Ding, & Wang, 2008) may have contributed to the differences in shrub communities between the two habitats, as most of the alpine shrub sites were distributed near the three river sources in the study (Figure 1).

### 4.2 Differences in the phylogenetic compositions of the shrub communities between the two habitats

Examining the differences in phylogenetic community composition can provide insights into the phylogenetic diversity and phylogenetic community structure (Aldana et al., 2017; Carlucci et al., 2017). Carlucci et al. (2017) argued that a community with more plant clades tends to have a higher level of phylogenetic diversity with phylogenetic overdispersion. In our study, a comparison of the PCPS I scores between the desert and alpine shrub communities indicated a significant difference between the phylogenetic compositions of these communities (Figure 6). The PCPS also revealed that the alpine shrub community was well represented by eudicots, monocots, and magnoliids, whereas the desert shrub community was largely represented by eudicots (Figure 7). Although the great differences in species richness between the two habitats (Figure S2) might lead to large differences in phylogenetic diversity and community structure, this conclusion remains unverified because the evolutionary history of a species can alter the relationship between species richness and phylogenetic diversity (Rodrigues & Gaston, 2002). For example, phylogenetic clustering occurs even in plant communities rich in closely related species. Therefore, the results of our phylogenetic composition analysis provide straightforward and solid evidence supporting the existence of differences in the phylogenetic diversity and phylogenetic community structure between the two habitats.

In summary, we observed that environmental factors had a stronger effect than climatic oscillations on shrub community assemblage and that species dispersal may help to increase the differences between shrub communities in two habitats. Our assessment of the phylogenetic community composition greatly elucidates the phylogenetic community structures of shrub communities on the northeastern QTP. As our research area was limited to the northeastern QTP, future studies over larger areas of this region, or even the entire QTP, are needed to gain a comprehensive understanding of the assemblages of the shrub communities on this plateau.

## CONFLICT OF INTEREST

The authors have no conflict of interest.

## AUTHOR CONTRIBUTION

**Yuanming Xiao:** Conceptualization (lead); Data curation (lead); Formal analysis (lead); Funding acquisition (supporting); Investigation (lead); Methodology (lead); Project administration (supporting); Resources (lead); Software (lead); Supervision (lead); Validation (lead); Visualization (lead); Writing‐original draft (lead); Writing‐review & editing (lead). **Lucun Yang:** Conceptualization (supporting); Data curation (supporting); Formal analysis (supporting); Funding acquisition (supporting); Investigation (supporting); Methodology (supporting); Project administration (supporting); Resources (supporting); Software (supporting); Supervision (supporting); Validation (supporting); Visualization (supporting); Writing‐original draft (supporting); Writing‐review & editing (supporting). **Xiuqing Nie:** Conceptualization (supporting); Data curation (supporting); Formal analysis (supporting); Funding acquisition (supporting); Investigation (supporting); Methodology (supporting); Project administration (supporting); Resources (supporting); Software (supporting); Supervision (supporting); Validation (supporting); Visualization (supporting); Writing‐original draft (supporting); Writing‐review & editing (supporting). **Changbin Li:** Conceptualization (supporting); Data curation (supporting); Formal analysis (supporting); Funding acquisition (supporting); Investigation (supporting); Methodology (supporting); Project administration (supporting); Resources (supporting); Software (supporting); Supervision (supporting); Validation (supporting); Visualization (supporting); Writing‐original draft (supporting); Writing‐review & editing (supporting). **Feng Xiong:** Conceptualization (supporting); Data curation (supporting); Formal analysis (supporting); Funding acquisition (supporting); Investigation (supporting); Methodology (supporting); Project administration (supporting); Resources (supporting); Software (supporting); Supervision (supporting); Validation (supporting); Visualization (supporting); Writing‐original draft (supporting); Writing‐review & editing (supporting). **Lingling Wang:** Conceptualization (supporting); Data curation (supporting); Formal analysis (supporting); Funding acquisition (supporting); Investigation (supporting); Methodology (supporting); Project administration (supporting); Resources (supporting); Software (supporting); Supervision (supporting); Validation (supporting); Visualization (supporting); Writing‐original draft (supporting); Writing‐review & editing (supporting). **Guoying Zhou:** Conceptualization (equal); Data curation (equal); Formal analysis (equal); Funding acquisition (lead); Investigation (lead); Methodology (equal); Project administration (lead); Resources (lead); Software (supporting); Supervision (lead); Validation (equal); Visualization (equal); Writing‐original draft (equal); Writing‐review & editing (equal).

## Supporting information

Fig S1

Fig S2

Fig S3

Table S1

Appendix S1

## Data Availability

We had uploaded our data to the Dryad at https://doi.org/10.5061/dryad.ngf1vhhr2
