# Supplementary figures and images for: Examining differences in phylogenetic composition enhances understanding of the phylogenetic structure of the shrub community in the northeastern Qinghai‐Tibetan Plateau
Source: Ecol Evol. 2020 Jun 8;10(13):6723–31. doi: 10.1002/ece3.6402 (PMC7381756; doi:10.1002/ece3.6402)

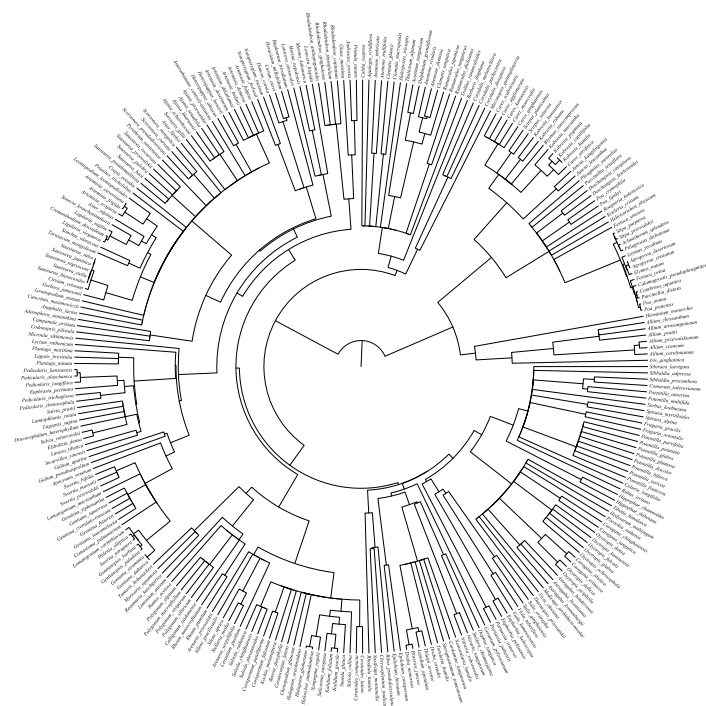

Supplement: Supplementary file 1 — Fig S1 [file ECE3-10-6723-s001.pdf]

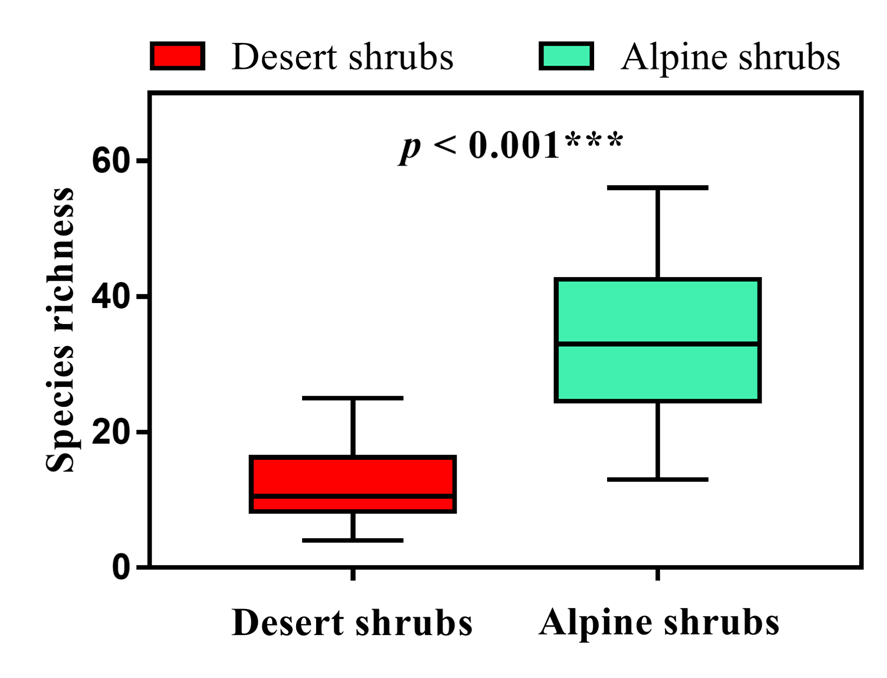

Supplement: Supplementary file 2 — Fig S2 [file ECE3-10-6723-s002.tif]

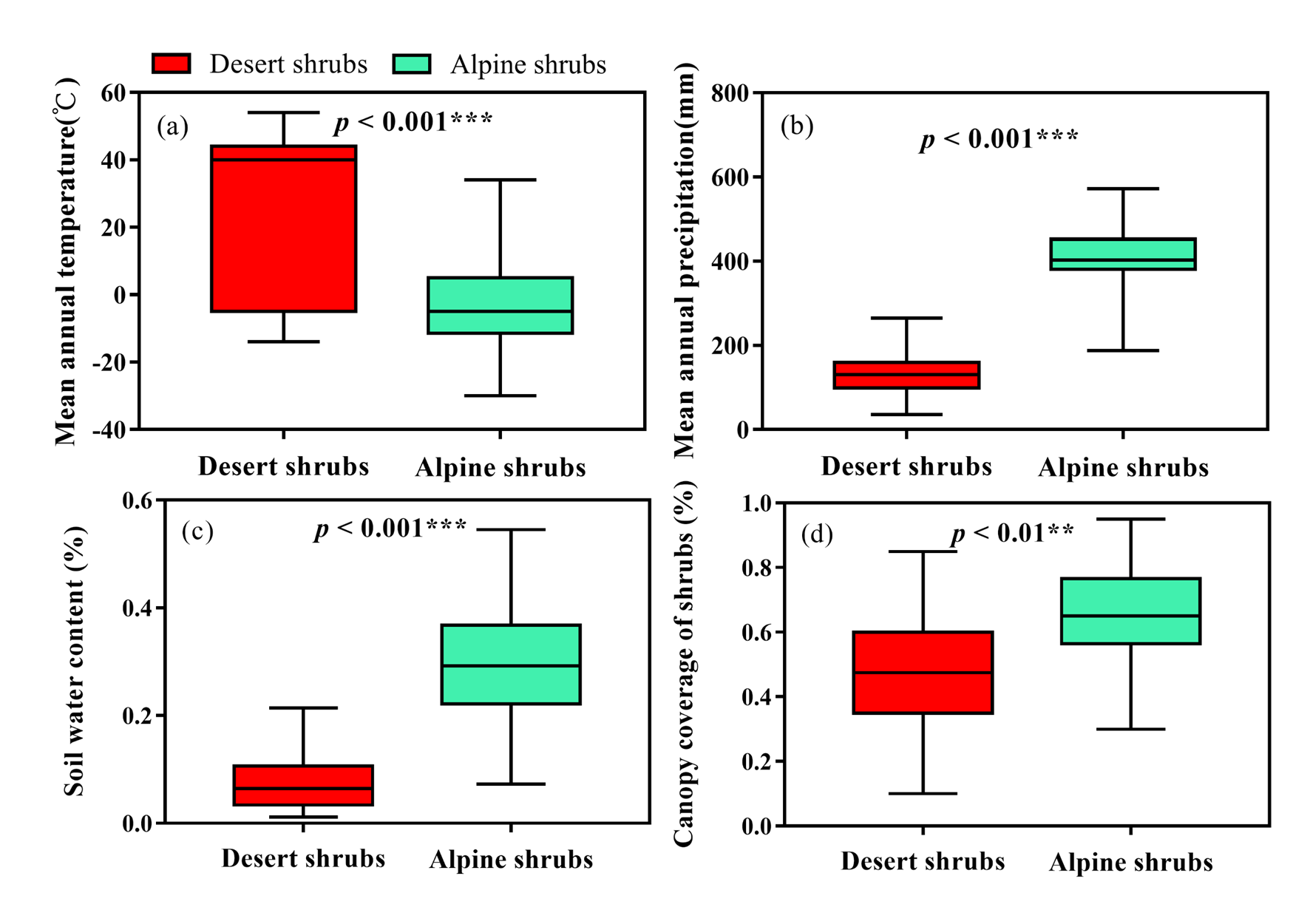

Supplement: Supplementary file 3 — Fig S3 [file ECE3-10-6723-s003.tif]
