# Supplementary material for: Examining differences in phylogenetic composition enhances understanding of the phylogenetic structure of the shrub community in the northeastern Qinghai‐Tibetan Plateau
Source: Ecol Evol. 2020 Jun 8;10(13):6723–31. doi: 10.1002/ece3.6402 (PMC7381756; doi:10.1002/ece3.6402)
Supplement: Supplementary file 5 — Appendix S1 [file ECE3-10-6723-s005.docx]

Supporting information for manuscript entitled: Examining differences in phylogenetic composition enhances understanding of the phylogenetic structure of the shrub community in the northeastern Qinghai-Tibetan Plateau

YuanMing Xiao^1,4^, LuCun Yang^1,2,3^, XiuQing Nie^1,4^, ChangBin Li^1,4^, Feng Xiong^1,4^, LingLing Wang^1,4^,GuoYing Zhou^1,2,3^

^1^ Northwest Institute of Plateau Biology, Chinese Academy of Sciences, Xining 810008, China

^2^ Key Laboratory of Tibetan Medicine Research, Chinese Academy of Sciences, Xining 810008, China

^3^ Qinghai Key Laboratory of Qinghai-Tibet Plateau Biological Resources, Xining 810008, China

^4^ University of Chinese Academy of Sciences, Beijing 100049, China

Author for correspondence: GuoYing Zhou

Tel: +86-971-6159630

Fax: +86-971-6143282

E-mail: [zhougy@nwipb.cas.cn](mailto:zhougy@nwipb.cas.cn)

ORCID: https://orcid.org/0000-0003-2485-6172

Address: 23# Xinning Road, Xining, Qinghai, P. R. China 810008


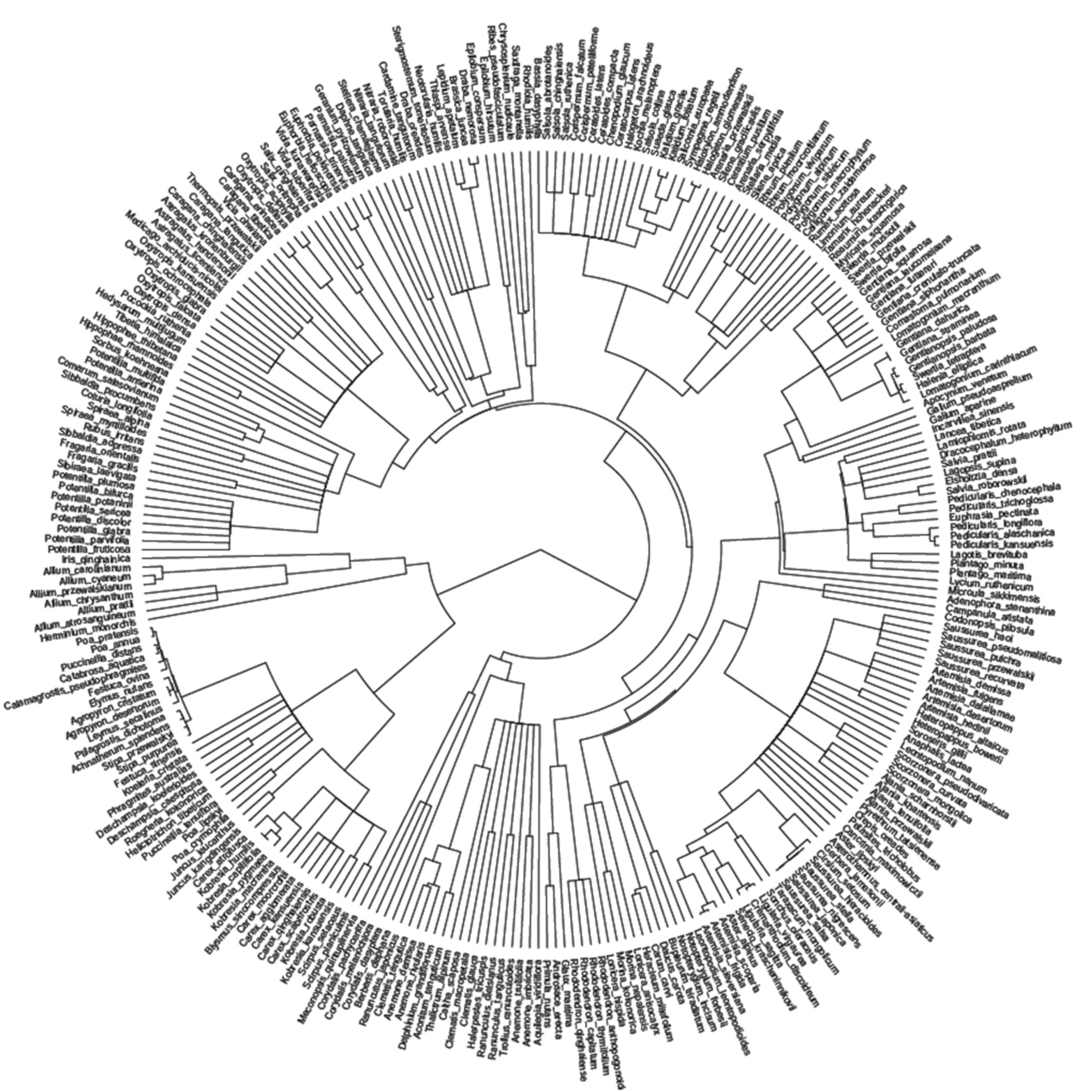


**FIGURE S1** Phylogenetic tree used in our study containing 285 species was constructed with Phylomatic based on mega-tree published by Zanne et al. (2014).


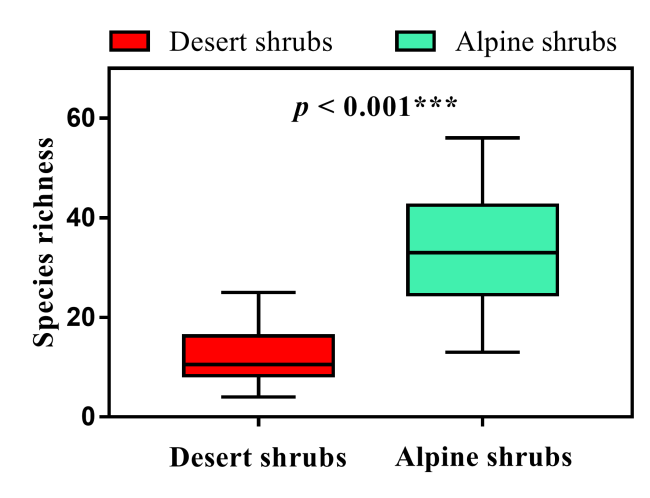


**FIGURE S2** Difference in species richness for shrub communities from different habitats on the northeastern of the Qinghai-Tibetan Plateau. The significant difference in species richness between the two habitats was determined by the Wilcoxon test.


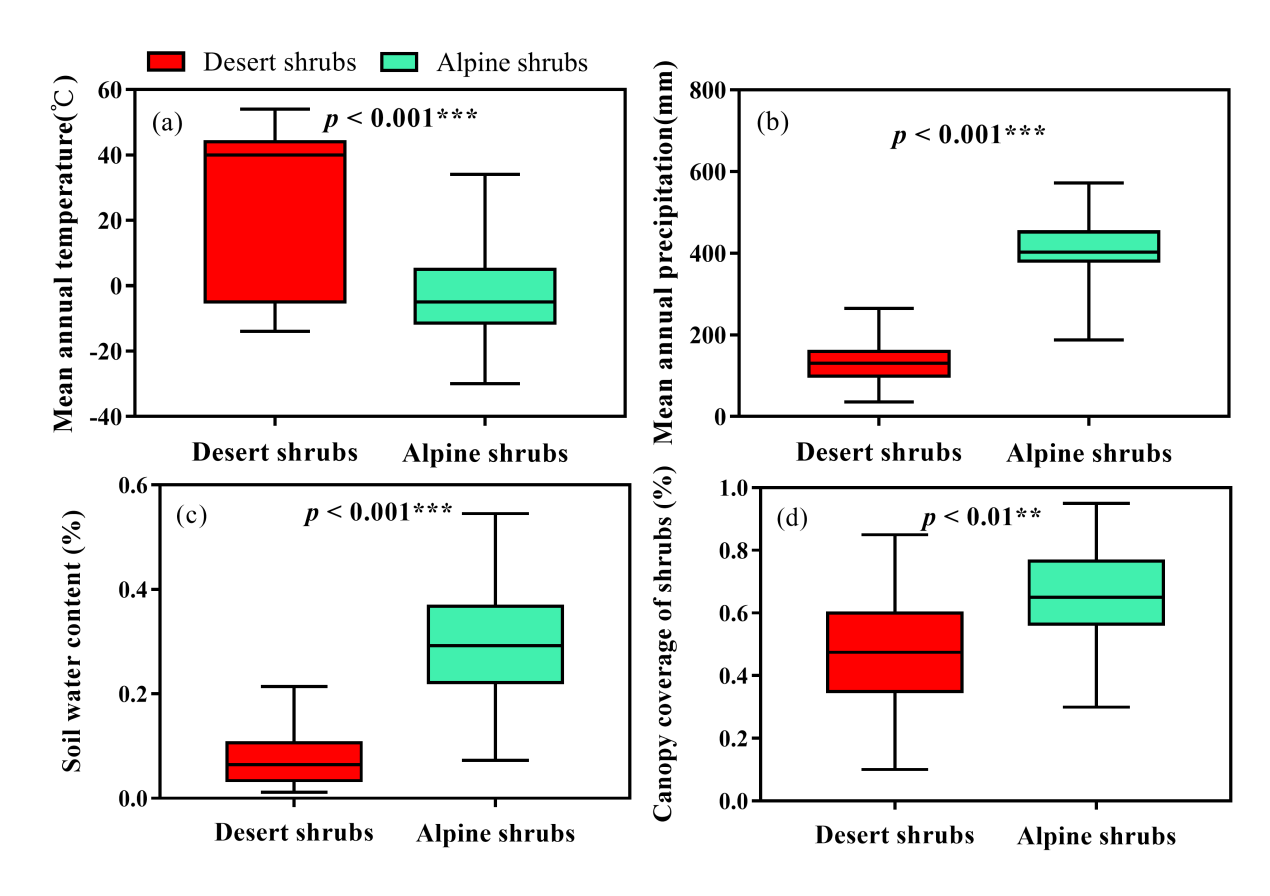


**FIGURE S3** Differences in Mean annual temperature (a), Mean annual precipitation (b), Soil water content (c) and Canopy coverage of shrubs (d) between desert shrubs and alpine shrubs in the northeastern Qinghai-Tibetan Plateau. The significant difference between the two habitats was determined by the Wilcoxon test.

**Table S1** Community species composition information of 61 sampling sites according to the APGIII (Angiosperm Phylogeny Group), which is basic information for construction of phylogenetic tree used in the study (**Seen in excel file**).

**Table S2** Pearson’s correlation between the first and second PCPS axis and value of the NRI.

|  | t | d.f. | r | p-values |
| --- | --- | --- | --- | --- |
| PCPS I | −3.15 | 59 | − 0.38 | 0.0026 |
| PCPS II | 2.07 | 59 | 0.26 | 0.0429 |

Note: for this analysis, we only used the NRI index to represent the phylogenetic community structure.
